# Alterations in lower limb kinematics and moments in partial foot amputation versus diabetic neuropathy

**DOI:** 10.1186/s13018-025-06376-w

**Published:** 2025-12-10

**Authors:** Omar M. Elabd, Bassem Galal Eldein El Nahass, Mona Mohamed Ibrahim

**Affiliations:** 1 Department of Physical Therapy for Orthopedics and Its Surgeries, Faculty of Physical Therapy, Delta University for Science and Technology, Gamsa, Menoufia Egypt; 2 Department of Physical Therapy for Musculoskeletal Disorders and Its Surgery, Faculty of Physical Therapy, Cairo University, Cairo, Egypt

**Keywords:** Kinematic, Joint moment, Partial foot amputation, Diabetic neuropathy

## Abstract

**Background:**

Gait compensatory mechanisms associated with partial foot amputation (PFA) and peripheral neuropathy (PN) aren’t well understood.

**Purpose:**

Current study aimed to assess deviations in the sagittal plane kinematics and moments of the lower limb joints in PFA due to PN versus PN alone.

**Methods:**

Sagittal plane ROM and moment of the ankle, knee, and hip joints were measured for 53 participants assigned into two well-matched groups: (A) 25 subjects with healed unilateral PFA and (B) 28 subjects with PN peripheral neuropathy (PN). Gait analysis was conducted using a baropodometric system and STT 3DMA system.

**Results:**

MANOVA revealed that both groups had a similar pattern of sagittal ROM curves of lower limbs (*p* = 0.402). However, PFA group showed a reduction in ankle plantar flexion during the preswing (*p* = 0.005). Descriptive analysis of the moment curves revealed that both groups had similar compensatory patterns, specifically reduction in ankle plantar flexion moment and reversal of knee moment during late stance. However, PN group had higher values.

**Conclusion:**

Individuals with either PFA due to PN or PN alone showed similar alterations in the sagittal plane kinematics and moments of the lower limb joints; they walked cautiously with excessive dorsiflexion throughout the stance phase, and the late stance phase was the most affected, while they compensated for the reduction in the ankle plantar moment by shifting the knee moment into extension moment. The results suggested that PN, not PFA, may be the primary cause of the gait alterations and PFA surgery only worsens the compensatory mechanisms.

## Introduction

Partial foot amputation (PFA) is a surgical procedure that involves the removal of part of the forefoot or mid-foot due to severe injury, infection, or physiological foot dysfunction, such as peripheral neuropathy (PN). It can lead to foot dysfunction due to anatomical loss of a part of peripheral body system that transmits body weight to the ground [1–5]. PN, a condition characterized by the loss of protective sensation in the foot, can cause injury and atrophy of intrinsic foot muscles and increase the risk of PFA due to a cascade of its complications [5–10]. The prevalence of diabetes has almost doubled since the 1980s, leading to an increase in the number of people requiring PFA surgery [11, 12].

Individuals with PN and PFA may have musculoskeletal impairments that develop gait deviations [1, 6]. Earlier studies on gait in patients with PN had limited variables and did not identify specific joint contributions, resulting in mixed findings [13–15]. Knowledge about PFA gait was primarily based on theoretical analysis and amputation impact speculations until limited studies began in the 1990s, a limited number of studies began to investigate PFA gait [16]. Most of these studies focused on transmetatarsal amputation especially while using prosthesis [17–20]. Nonetheless, these studies had methodological flaws, such as an insufficient sample size and inadequate reporting of procedures [16].

Addressing the limited understanding of compensatory mechanisms, previous contradictory results, and methodological flaws and aiming to fill up that knowledge gap, a comprehensive gait analysis of patients with PFA due to PN complications and those with PN alone has been conducted. The prior papers revealed that both groups had similarities in compensatory mechanisms regarding spatiotemporal characteristics, ground reaction force (GRF), and loading patterns. They had cautious walking, less pronounced peaks of plantar pressure and GRF, and posterior body shifting to compensate for insufficient forefoot support, hypothesizing that the systemic pathology of PN causes these alterations, with PFA worsening the compensatory mechanism [21, 22].

However, the changes in the range of motion (ROM) and joint moment of the lower limb joints that could cause these gait alterations, as revealed in the prior papers, remained unclear. Therefore, the purpose of the current study is to answer the question, “What are the changes in the sagittal plane kinematics and moments of lower limb joints during gait in PFA versus PN that could cause these gait alterations?” Answering that question will help to specify the compensatory mechanisms secondary to such disorders, leading to more effective rehabilitation, improving gait function and quality of life, and preventing complications and further amputations for individuals with PN and PFA.

## Methods

The current study is a part of a comprehensive prospective cohort observational research, carried out in accordance with the 1964 Helsinki Declaration's ethical criteria and registered with Clinicaltrials.gov (NCT05161364), to explore the changes in gait mechanics in PFA secondary to PN complications compared to PN patients without amputation, to accurately specify gait compensatory mechanisms for this population. The Ethical Committee for Human Research at Cairo University's Faculty of Physical Therapy, Egypt, approved the study (NO: P.T.REC/012/002730) [21, 22].

### Participants

Physicians or orthopedic surgeons invited their patients with PN diagnosis, with/without PFA, to take part in the current study. The study included participants of both genders with ages ranging from 40 to 70. PN subjects with grade I and II were included. PFA subjects with fully healed PFA were included. In order to prevent heterogeneity from confounding the results, the amputation level was restricted to the forefoot. Every participant was able to walk on their own to carry out their daily living activities. Exclusion criteria included acute inflammation symptoms, bilateral PFA, active foot problems, and amputation levels above the MTP joint. After completing a written consent form, participants were grouped into two groups to take part in the study. Subjects in the PFA group (A): had healed unilateral PFAs secondary to PN, while the PN group (B) had PN with neither ulcer nor amputation. The prior paper (Elabd et al., 2023) provided further details on participant diagnosis, recruitment, and allocation.

### Assessment tools

The gait analysis was carried out in the motion analysis lab of Delta University for Science and Technology. The study was conducted using baropodometric system comprised of FREEMED platform model 160 × 40 and FREESTEP software V.2.01.001 (Sensor Medica, Inc., Via Umberto Agnelli 11, 00012 Guidonia Montecelio, Rome, Italy—Tel: + 390774356165, Email: info@sensormedica.com), as well as STT 3DMA system comprised of CLINICAL 3DMA V.2022.0 and STT Helen Hayes protocol with 15 reflective markers as shown in Table 1 (STT Systems Company, Zuatzu Business Park, Easo Building, 2nd Floor, San Sebastián, Spain—Phone: (+ 34) 943 31 77 77, Fax: (+ 34) 943 31 64 31, Email: info@stt-systems.com) and 4 OptiTrack cameras moel Flex 13 (NaturalPoint, Inc., U.S.A., Phone: + 1-541-753–6645, Fax1-541-753-6689).

**Table 1:** Helen Hayes marker placement (Kadaba et al., 1990)

Anatomical position of markers	
1	On the right anterior superior iliac spine
2	On the left anterior superior iliac spine
3	On the right lateral thigh with an extender, just below the swing of the hand
4	On the left lateral thigh with an extender, just below the swing of the hand
5	On the right-knee lateral epicondyle of the right femur
6	On the left-knee lateral epicondyle of the left femur
7	On the right calf with an extender
8	On the left calf with an extender
9	On the right lateral malleolus along an imaginary line that passed through the transmalleolar axis
10	On the left lateral malleolus along an imaginary line that passed through the transmalleolar axis
11	Between the second and the third metatarsal of the right foot, approximately over the Metatarso-phalangeal joint
12	Between the second and the third of the left foot, approximately over the Metatarso-phalangeal joint
13	On the L5-Sacrum joint
14	On the right greater trochanter
15	On the left greater trochanter

### Kinematic assessment

**Fig. 1:**
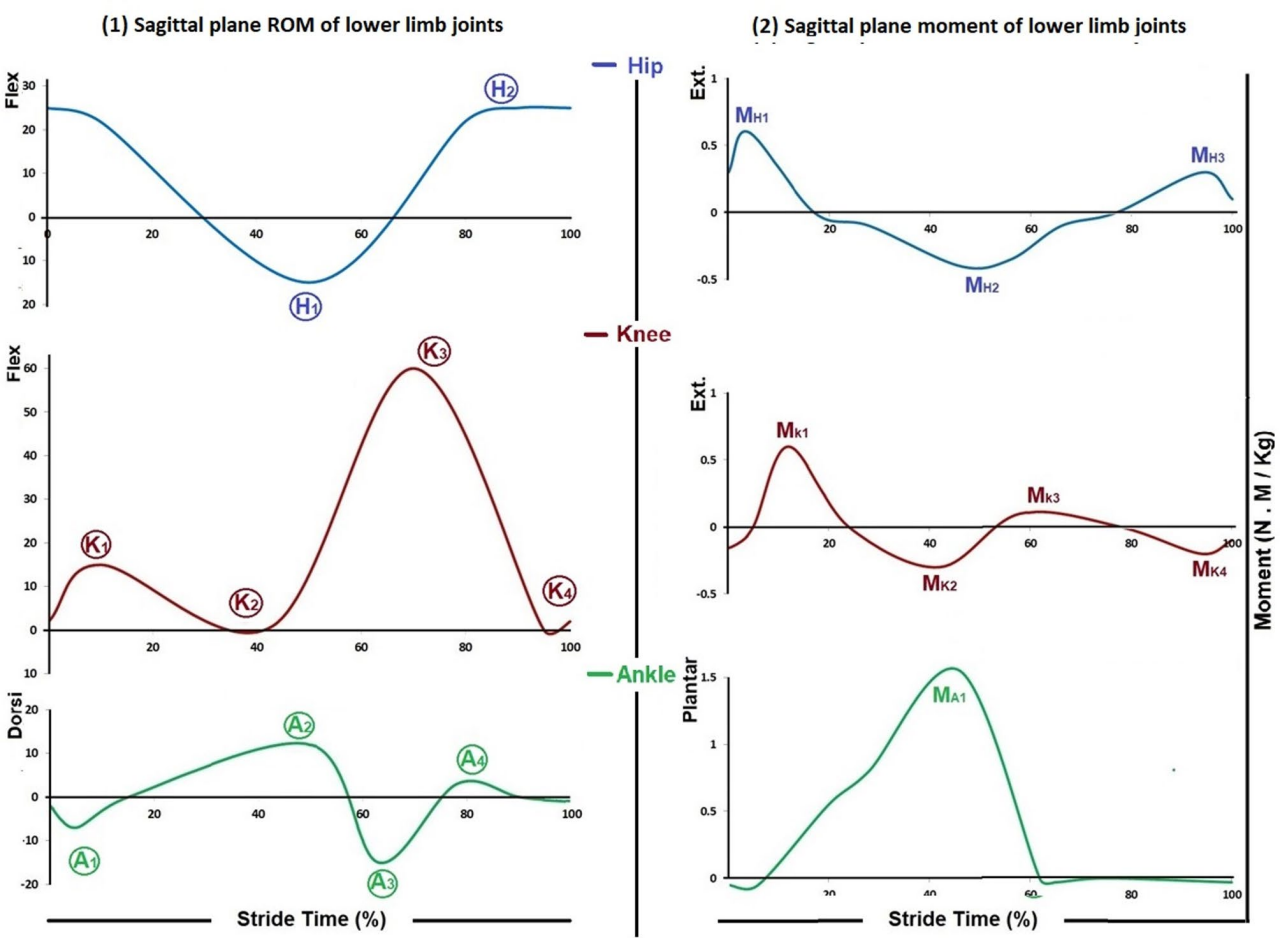
(1) ROM and (2) moment of lower limb joints in the sagittal plane; (Blue) the hip joint whereas, (H_1_) and (H_2_) the peaks of the ROM curve and (M_H1_), (M_H2_), and (M_H3_) the peaks of the moment curve; (Red) the knee joint whereas, (K_1_), (K_2_), (K_3_), and (K_4_) the peaks of the ROM curve and (M_K1_), (M_K2_), (M_K3_), and (M_K4_) the peaks of the moment curve; and (Green) the ankle joint whereas, (A_1_), (A_2_), (A_3_), and (A_4_) the peaks of the ROM curve and (A_K1_) the peak of the moment curve (Adapted from Winter, 1988; Janet & Cerny, 2018)

CLINICAL 3DMA software was used to measure speed and angular displacement of the pelvis, ankle, knee, and hip joints using the STT Helen Hayes protocol. Walking speed was measured from the recorded strides in m/s. The sagittal plane angular displacement (angle projected in the sagittal plane) of the ankle, knee, and hip of the successful strides was plotted as curves by Microsoft Excel V.14.0 (Office 2010, Microsoft Corporation), assuming that angular displacement in a counter-clockwise direction has a + ve polarity. Considering the normal pattern of the curves, peaks where the curve changes its direction were identified to represent the change in the ROM of the lower limb joints during the gait cycle (GC). They were A_1_, A_2_, A_3_, and A_4_ for the ankle joint during loading response, terminal stance, preswing, and midswing, respectively; K_1_, K_2_, K_3_, and K_4_ for the knee joint during loading response, terminal stance, initial swing, and terminal swing, respectively; and H_1_ and H_2_ for the hip joint during terminal stance and midswing (Fig. 1–1). The peak magnitude and time were measured in degrees and percentage of stride time, respectively.

### Gait analysis procedures

**Fig. 2:**
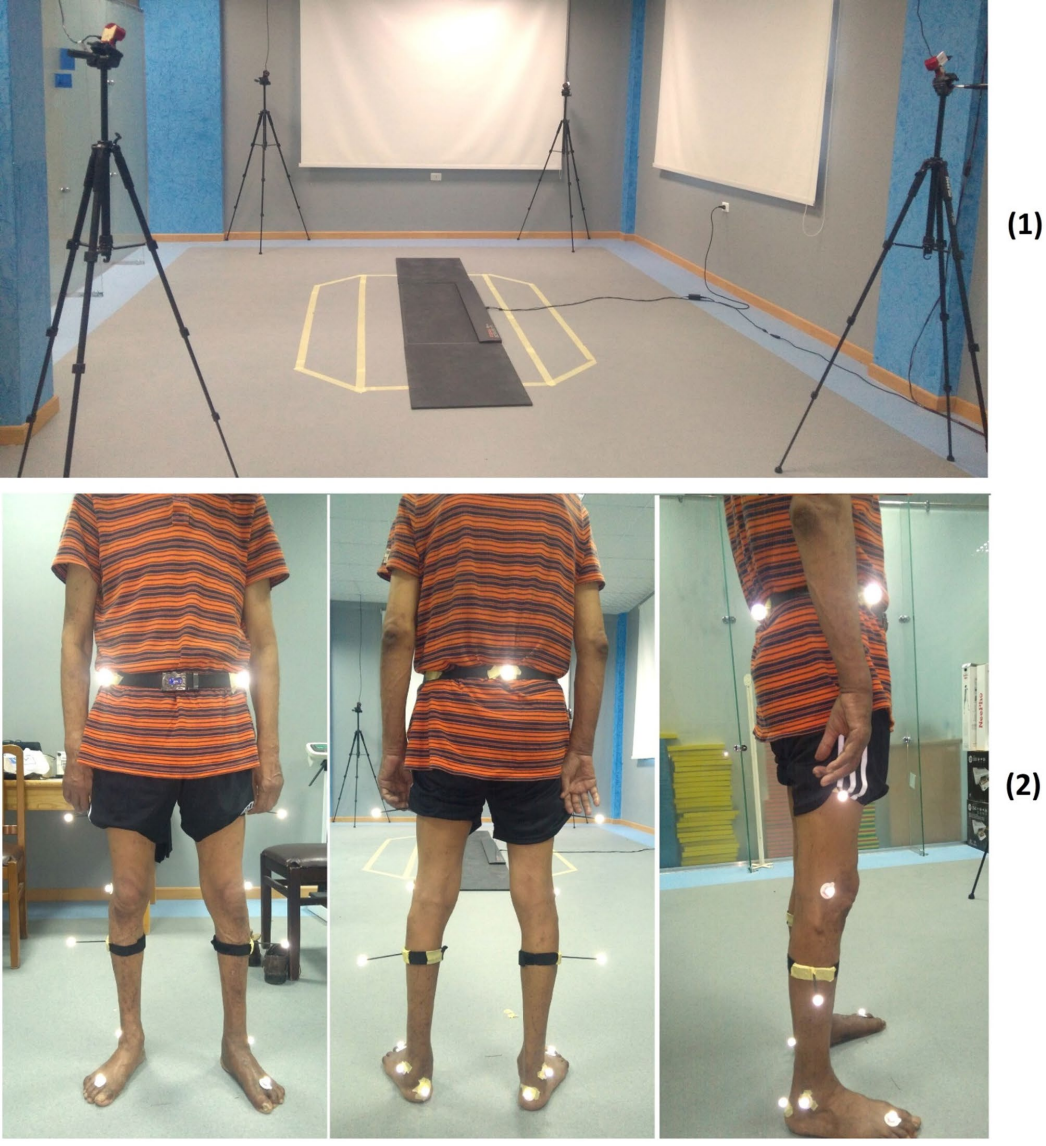
(1) Motion analysis lab arrangement and (2) Marker placement on the patient’s lower limb

Regarding the STT 3DMA system, the cameras were set up with a rate of 30 FPS and a 1/3-inch sensor size and arranged in a standard configuration. The FREEMED platform was placed over level ground inside the capture area of the STT 3DMA (Fig. 2–1). It was set and calibrated at 10 bit auto, XY resolution at 2.5 dpi, Z resolution at 8 bit, max pressure at 150 N/cm^2^, and frequency at 120 fps.

Prior to each assessment session, the STT 3DMA system calibration was checked. If the result was deemed correct, the assessment session would begin; otherwise, recalibration would be performed. The participants were asked to take off their shoes and were equipped with 15 reflective markers according to the STT Helen Hayes Protocol for the lower limb [23] (Fig. 2–2), and were instructed to walk barefoot for as many steps as needed to get comfortable with the testing environment. As soon as the participant was comfortable with the testing environment, the session was recorded and saved. At the end of the recording session, the recorded data from both the CLINICAL 3DMA and FREESTEP software were verified and filtered. The recorded strides were checked, and unsuccessful strides were discarded. Further details on assessment instruction and checking process of the FREESTEP data were previously described [21].

The CLINICAL 3DMA software exports the marker coordinates and the angular displacement of the pelvis, hip, knee, and ankle joints to a CSV file, and the speed data is exported to a PDF file for each patient. The FREESTEP software exports the COP and GRF data for each subject to a CSV file. The mean of three successful trials was calculated for each outcome as the peak values of each trial detected and then averaged.

### Joint moment assessment

**Fig. 3:**
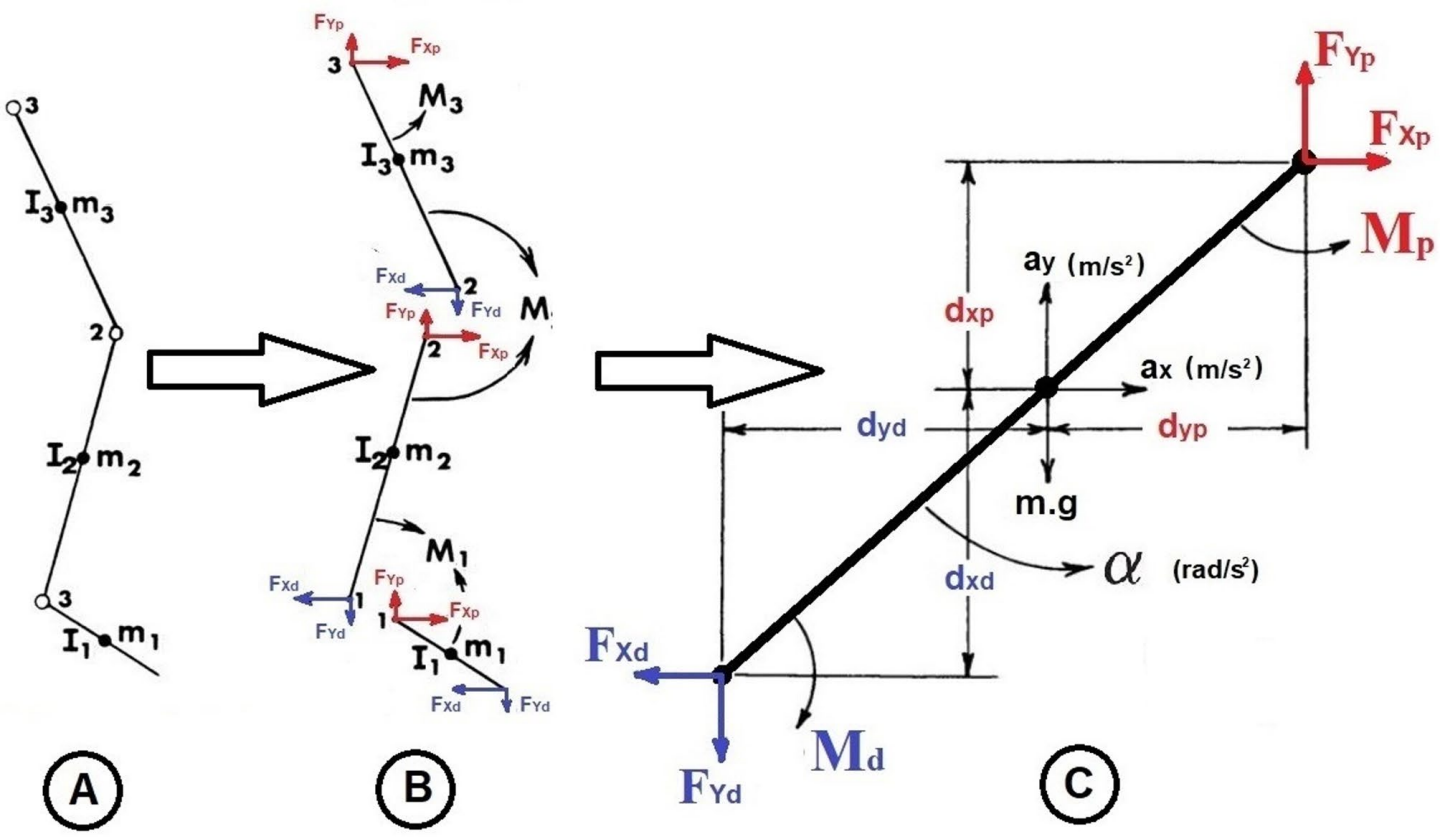
Inverse dynamics; **A** link-segment model, **B** Free-body diagram **C** complete free-body diagram of a single segment (Adapted from Winter, 2009)

The sagittal plane moments of the lower limb joints were calculated using inverse dynamics based on a free-body diagram of the link-segment model of the lower limb, which relied on Newton’s third law (Fig. 3) [24]. The final equations for the calculation of the joint moment of the ankle, knee, and hip joints were:

1 $$ {\mathrm{M}}_{{{\mathrm{Ankle}}}} = \, \Sigma {\mathrm{M}}_{{{\mathrm{foot}}}} - {\text{ M}}_{{\text{p foot}}} + {\text{ M}}_{{\text{d foot}}} $$

2 $$ {\mathrm{M}}_{{{\mathrm{Knee}}}} = \, \Sigma {\mathrm{M}}_{{{\mathrm{shank}}}} - {\text{ M}}_{{\text{p shank}}} + {\text{ M}}_{{\text{d shank}}} + {\mathrm{M}}_{{{\mathrm{Ankle}}}} $$

3 $$ {\mathrm{M}}_{{{\mathrm{Hip}}}} = \, \Sigma {\mathrm{M}}_{{{\mathrm{thigh}}}} - {\text{ M}}_{{\text{p thigh}}} + {\mathrm{M}}_{{\text{d thigh}}} + {\mathrm{M}}_{{{\mathrm{Knee}}}} $$

whereas M: joint moment, ΣM: the summation of the moments, M_p_: the moment at the proximal end of a joint, and M_d_: the moment at the distal end of a joint.

The anthropometric measurements of the foot, shank, and thigh, full kinematic variables of each segment in absolute coordinates, GRF, and location of COP were input data for these calculations [24]. The full kinematic variables of each segment in absolute coordinates were directly measured by the CLICAL 3DMA. The GRF in the plane of progression and the location of the COP were directly measured by the FREEMED platform. The anthropometric measurements were based on total body height and mass measured in meters and kilograms, respectively, according to [25–27]. The calculated moment was normalized to the body mass and expressed in N.m/kg. The calculation process was conducted using Microsoft Excel software. Considering the normal pattern of the curves, their peaks, where the curve changes its direction, were identified to represent the change in the moment of the lower limb joints during the GC (Fig. 1–2).

### Statistical analysis

The reported data were analyzed using IBM SPSS Statistics (Version 26). The right limb of the PN group was selected to be compared to the amputated limb of the PFA as there were no significant differences between the right and left sides (Elabd et al., 2023). The recorded data for the recorded kinematic variables for all subjects were analyzed for normality using the Shapiro–Wilk test. The studied variables didn’t significantly violate the assumption of the normality distribution. Multivariate analysis of variance (MANOVA) was used to compare the amputated limb of the PFA group with the right limb of the PN group for the measured kinematic variables. The level of significance for all tests was set at a *p*-value ≤ 0.05.

Descriptive statistics (mean and standard deviation, and upper and lower bounds at a 95% confidence interval) of the moment curves were calculated and presented for each group.

## Results

Fifty three subjects were included in the multivariate test out of one hundred sixty-seven screened for eligibility criteria. They were allocated into two groups: (A) the PFA group: 25 subjects with healed unilateral PFAs due to PN; and (B) the PN group: 28 subjects with PN without amputation. The PFA and PN groups were well-matched demographically (*p* = 0.103). Further details on participant recruitment, retention, and analysis of their demographic characteristics were previously detailed in the previous work (Elabd et al., 2023). This study analyzed the speed and the sagittal plane kinematics and moments of the ankle, knee, and hip joints for both the PFA and PN groups.

**Fig. 4:**
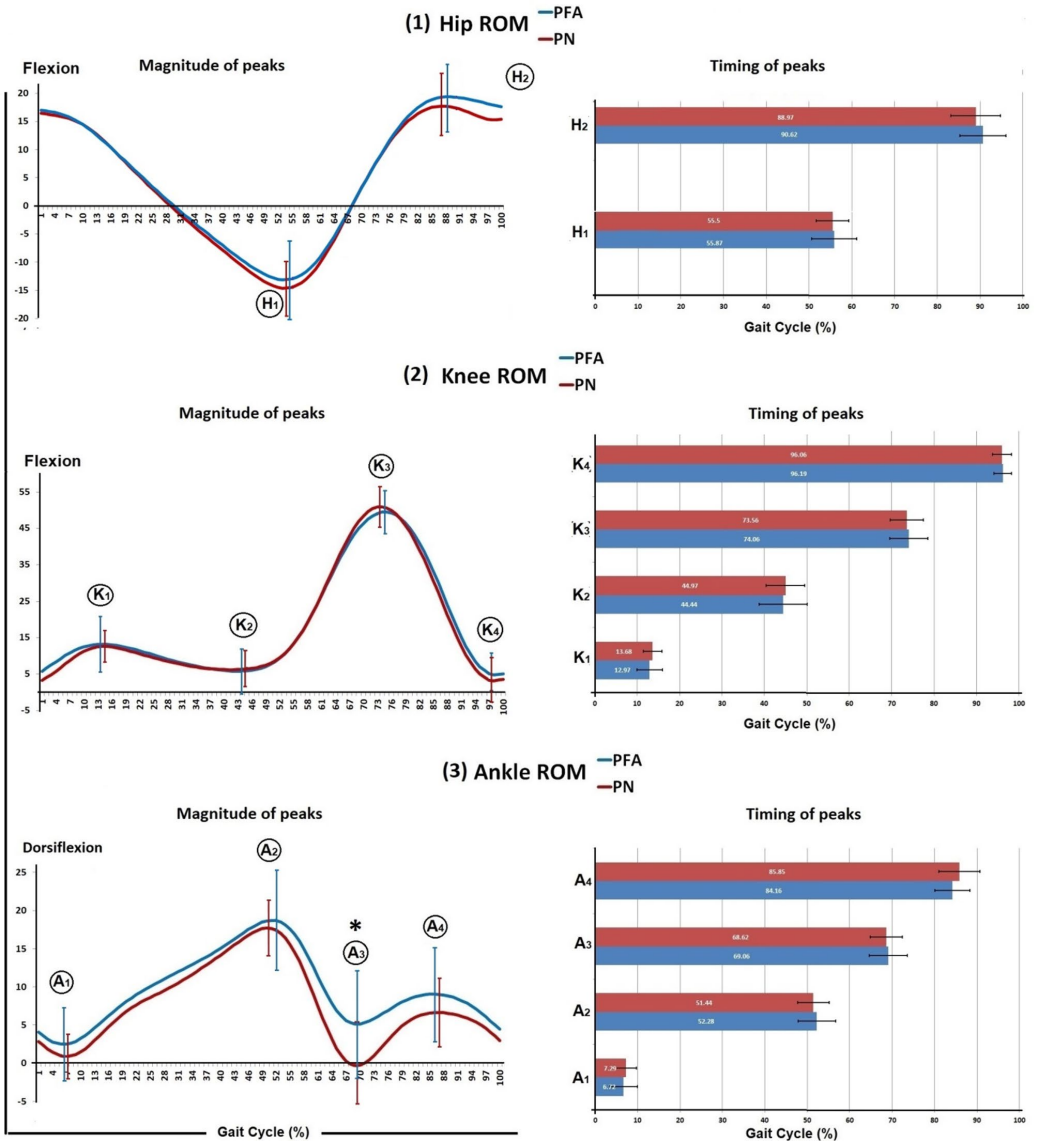
Sagittal plane kinematics of lower limb joints; (1) Hip ROM whereas, (H_1_) and (H_2_) the peaks of the; (2) Knee ROM whereas, (K_1_), (K_2_), (K_3_), and (K_4_) the peaks of the curve; and (3) Ankle ROM whereas, (A_1_), (A_2_), (A_3_), and (A_4_) the peaks of the curve, mean and standard deviation to the left, and peak timing to the right

Multivariate analysis of variance (MANOVA) was conducted and presented in Table 2. The MANOVA revealed that both groups had similar pattern of kinematic curves without an overall significant difference (Wilks’ Lambda = 0.660, *F* = 1.080, and *p* = 0.402). Furthermore, the PFA group showed a reduction in the third peak, ankle plantar flexion during the preswing subphase, compared to the PN group (*p* = 0.005). All other measured kinematic variables showed no significant differences between the two groups (*p* > 0.05) (Table 2, Fig. 4).

**Table 2:** Multivariate comparison for the measured kinematic variables between the PFA and PN groups

Variables		PN (Right limb) N = 28		PFA (Amputated limb) N = 25		Mean difference	95% confidence interval of the mean difference		*P* value
		Mean	SD	Mean	SD		Lower	Upper	
Walking Speed (m/s)		0.81	0.18	0.77	0.20	0.04	− 0.05	0.13	0.385
Ankle Sagittal plane ROM	First peak (degrees)	0.18	3.06	1.30	4.85	− 1.11	− 3.09	0.87	0.266
	Second peak (degrees)	18.81	3.77	19.66	6.58	− 0.85	− 3.47	1.77	0.519
	Third peak (degrees)	− 2.46	5.73	2.14	7.09	− 2.40	− 5.09	0.29	0.005*
	Forth peak (degrees)	7.46	4.58	9.86	6.27	− 4.60	− 7.76	− 1.43	0.079
	Time of first peak (% of stride)	7.29	2.41	6.72	3.25	− 0.44	− 2.48	1.59	0.414
	Time of second peak (% of stride)	51.44	3.71	52.28	4.37	0.58	− 0.82	1.97	0.402
	Time of third peak (% of stride)	68.62	3.78	69.06	4.48	− 0.83	− 3.92	2.25	0.664
	Time of forth peak (% of stride)	85.85	4.80	84.16	4.14	− 1.28	− 3.96	1.40	0.130
Knee Sagittal plane ROM	First peak (degrees)	12.96	4.39	13.80	7.80	− 0.84	− 2.83	1.15	0.592
	Second peak (degrees)	5.86	5.13	5.58	6.19	− 0.38	− 2.61	1.86	0.845
	Third peak (degrees)	54.23	5.76	53.33	5.78	− 0.13	− 1.18	0.93	0.527
	Forth peak (degrees)	2.16	6.24	3.72	5.57	− 0.50	− 2.56	1.55	0.290
	Time of first peak (% of stride)	13.68	2.23	12.97	2.96	− 1.56	− 4.47	1.36	0.274
	Time of second peak (% of stride)	44.97	4.59	44.44	5.71	1.70	− 0.51	3.91	0.676
	Time of third peak (% of stride)	73.56	3.89	74.06	4.46	0.90	− 1.93	3.74	0.626
	Time of forth peak (% of stride)	96.06	2.24	96.19	2.04	0.53	− 2.01	3.07	0.808
Hip Sagittal plane ROM	First peak (degrees)	− 15.63	5.34	− 14.36	5.54	0.71	− 0.57	1.99	0.345
	Second peak (degrees)	18.75	6.14	20.04	5.33	− 1.29	− 4.13	1.55	0.367
	Time of first peak (% of stride)	55.50	3.8	55.87	5.22	− 1.65	− 4.42	1.11	0.739
	Time of second peak (% of stride)	88.97	5.84	90.62	5.37	0.27	− 2.51	3.06	0.237

**Fig. 5:**
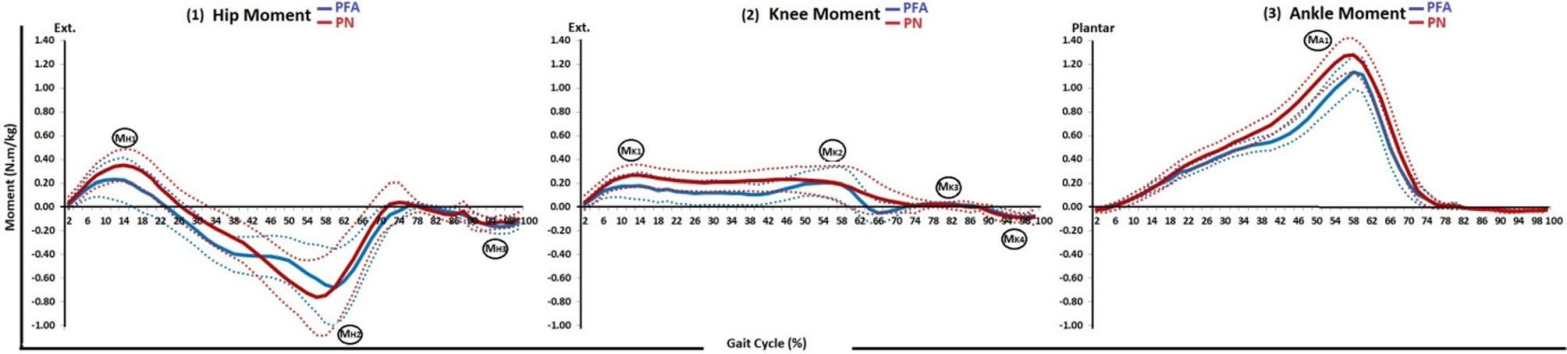
Mean and 95% Confidence interval of the sagittal plane moment of the lower limb joints of both groups; (1) hip joint whereas (M_H1_), (M_H2_), and (M_H3_) the peaks of the curve, (2) knee joint whereas (M_K1_), (M_K2_), (M_K3_), and (M_K4_) the peaks of the curve, and (3) ankle joint whereas (A_K1_) the peak of the curve

Descriptive analysis of the moment curves of the ankle, knee and hip joints for both groups was conducted and presented in Table 3. The peaks of the moment curves were identified at the point where the curve changes its direction Fig. 5. Overall, the moment curves of the ankle, knee and hip joints showed similar patterns for both groups. However, the PN group had higher peaks across the curves.

**Table 3:** Descriptive statistics of the joint moment of the lower limb joints (ankle, knee, and hip) for both the PN and PFA groups

% of Gait cycle	PN				PFA			
	Mean	SD	95% confidence interval		Mean	SD	95% confidence interval	
			Lower bound	Upper bound			Lower bound	Upper bound
Ankle joint moment in sagittal plane								
2	0.03	0.06	0.00	0.05	0.02	0.02	0.01	0.02
4	0.01	0.09	− 0.01	0.04	0.00	0.03	− 0.01	0.01
6	− 0.01	0.09	− 0.04	0.02	− 0.02	0.03	− 0.03	− 0.01
8	− 0.04	0.11	− 0.08	0.00	-0.04	0.03	-0.05	-0.03
10	− 0.07	0.13	− 0.12	− 0.03	− 0.07	0.04	− 0.09	− 0.06
12	− 0.11	0.14	− 0.16	− 0.07	− 0.11	0.06	− 0.13	− 0.09
14	− 0.16	0.16	− 0.21	− 0.10	− 0.16	0.12	− 0.21	− 0.12
16	− 0.21	0.18	− 0.27	− 0.15	− 0.20	0.11	− 0.24	− 0.16
18	− 0.26	0.19	− 0.33	− 0.20	− 0.23	0.11	− 0.27	− 0.19
20	− 0.32	0.20	− 0.38	− 0.25	− 0.29	0.17	− 0.35	− 0.23
22	− 0.36	0.20	− 0.43	− 0.30	− 0.30	0.12	− 0.35	− 0.26
24	− 0.40	0.20	− 0.47	− 0.33	− 0.34	0.13	− 0.38	− 0.29
26	− 0.44	0.21	− 0.50	− 0.37	− 0.37	0.13	− 0.42	− 0.32
28	− 0.46	0.21	− 0.54	− 0.39	− 0.41	0.13	− 0.45	− 0.36
30	− 0.50	0.22	− 0.58	− 0.43	− 0.44	0.14	− 0.49	− 0.39
32	− 0.54	0.23	− 0.62	− 0.47	− 0.47	0.15	− 0.52	− 0.42
34	− 0.58	0.24	− 0.66	− 0.49	− 0.49	0.15	− 0.55	− 0.44
36	− 0.62	0.25	− 0.70	− 0.53	− 0.52	0.16	− 0.58	− 0.46
38	− 0.65	0.25	− 0.74	− 0.57	− 0.53	0.17	− 0.59	− 0.47
40	− 0.69	0.27	− 0.78	− 0.60	− 0.54	0.19	− 0.61	− 0.48
42	− 0.75	0.28	− 0.84	− 0.66	− 0.58	0.21	− 0.65	− 0.50
44	− 0.81	0.29	− 0.91	− 0.72	− 0.62	0.23	− 0.70	− 0.53
46	− 0.89	0.31	− 0.99	− 0.78	− 0.67	0.25	− 0.76	− 0.58
48	− 0.97	0.33	− 1.08	− 0.86	− 0.74	0.29	− 0.84	− 0.64
50	− 1.05	0.35	− 1.17	− 0.93	− 0.83	0.33	− 0.94	− 0.71
52	− 1.13	0.40	− 1.27	− 1.00	− 0.91	0.37	− 1.04	− 0.78
54	− 1.21	0.43	− 1.36	− 1.07	− 0.99	0.40	− 1.13	− 0.85
56	− 1.27	0.45	− 1.42	− 1.12	− 1.07	0.40	− 1.21	− 0.93
58	− 1.28	0.42	− 1.42	− 1.14	− 1.13	0.41	− 1.27	− 0.99
60	− 1.21	0.43	− 1.36	− 1.07	− 1.11	0.43	− 1.26	− 0.96
62	− 1.07	0.48	− 1.24	− 0.91	− 0.94	0.42	− 1.08	− 0.79
64	− 0.90	0.50	− 1.07	− 0.74	− 0.72	0.41	− 0.86	− 0.58
66	− 0.69	0.48	− 0.85	− 0.53	− 0.50	0.43	− 0.64	− 0.35
68	− 0.46	0.43	− 0.60	− 0.32	− 0.32	0.40	− 0.46	− 0.19
70	− 0.28	0.34	− 0.39	− 0.17	− 0.19	0.31	− 0.30	− 0.08
72	− 0.14	0.24	− 0.22	− 0.06	− 0.11	0.23	− 0.19	− 0.03
74	− 0.06	0.17	− 0.12	0.00	− 0.06	0.18	− 0.13	0.00
76	− 0.02	0.10	− 0.06	0.01	− 0.02	0.10	− 0.06	0.01
78	− 0.01	0.06	− 0.03	0.01	0.00	0.04	− 0.02	0.01
80	− 0.01	0.06	− 0.03	0.01	− 0.01	0.09	− 0.04	0.02
82	0.01	0.02	0.00	0.01	0.01	0.01	0.00	0.01
84	0.01	0.02	0.01	0.02	0.01	0.01	0.01	0.02
86	0.02	0.03	0.01	0.03	0.01	0.01	0.01	0.02
88	0.02	0.02	0.01	0.03	0.02	0.01	0.02	0.02
90	0.03	0.04	0.02	0.04	0.03	0.01	0.02	0.03
92	0.03	0.04	0.02	0.05	0.03	0.01	0.03	0.03
94	0.03	0.04	0.02	0.05	0.03	0.01	0.03	0.04
96	0.03	0.03	0.02	0.04	0.03	0.01	0.03	0.04
98	0.03	0.03	0.02	0.04	0.03	0.01	0.02	0.03
100	0.02	0.06	0.00	0.04	0.02	0.02	0.02	0.03
Knee joint moment in sagittal plane								
2	0.04	0.18	− 0.02	0.10	0.03	0.07	0.00	0.05
4	0.10	0.20	0.04	0.17	0.08	0.13	0.04	0.13
6	0.17	0.19	0.10	0.23	0.13	0.17	0.07	0.19
8	0.22	0.23	0.14	0.30	0.16	0.22	0.09	0.24
10	0.24	0.27	0.16	0.33	0.17	0.26	0.08	0.27
12	0.26	0.28	0.17	0.36	0.17	0.30	0.07	0.28
14	0.26	0.27	0.17	0.36	0.18	0.33	0.07	0.30
16	0.25	0.27	0.16	0.34	0.16	0.32	0.05	0.28
18	0.24	0.25	0.15	0.32	0.14	0.30	0.04	0.25
20	0.23	0.25	0.15	0.31	0.15	0.28	0.05	0.25
22	0.22	0.24	0.14	0.30	0.13	0.29	0.03	0.23
24	0.22	0.24	0.14	0.30	0.12	0.29	0.02	0.23
26	0.21	0.24	0.13	0.29	0.12	0.29	0.02	0.22
28	0.21	0.23	0.13	0.28	0.12	0.29	0.02	0.22
30	0.21	0.23	0.13	0.29	0.12	0.30	0.02	0.22
32	0.21	0.23	0.13	0.29	0.12	0.28	0.02	0.22
34	0.21	0.23	0.13	0.29	0.11	0.28	0.01	0.21
36	0.21	0.24	0.13	0.30	0.11	0.28	0.02	0.21
38	0.22	0.25	0.13	0.30	0.10	0.26	0.01	0.20
40	0.22	0.27	0.13	0.31	0.10	0.25	0.02	0.19
42	0.23	0.28	0.13	0.32	0.12	0.25	0.03	0.20
44	0.23	0.29	0.13	0.33	0.13	0.25	0.04	0.22
46	0.23	0.30	0.13	0.33	0.15	0.26	0.06	0.24
48	0.23	0.31	0.13	0.34	0.18	0.27	0.08	0.27
50	0.23	0.33	0.12	0.34	0.20	0.28	0.10	0.29
52	0.22	0.35	0.11	0.34	0.20	0.31	0.09	0.31
54	0.21	0.37	0.09	0.34	0.20	0.34	0.09	0.32
56	0.20	0.42	0.06	0.35	0.20	0.38	0.07	0.34
58	0.19	0.47	0.03	0.35	0.19	0.42	0.04	0.34
60	0.16	0.51	− 0.01	0.34	0.14	0.43	− 0.01	0.29
62	0.13	0.51	− 0.04	0.30	0.06	0.40	− 0.08	0.20
64	0.10	0.47	− 0.06	0.26	− 0.01	0.34	− 0.13	0.10
66	0.08	0.39	− 0.05	0.21	− 0.05	0.27	− 0.14	0.04
68	0.06	0.31	− 0.05	0.16	− 0.04	0.21	− 0.11	0.03
70	0.04	0.26	− 0.05	0.13	− 0.02	0.14	− 0.07	0.03
72	0.03	0.21	− 0.05	0.10	0.00	0.08	− 0.02	0.03
74	0.01	0.18	− 0.05	0.07	0.01	0.05	0.00	0.03
76	0.02	0.11	− 0.02	0.06	0.02	0.03	0.02	0.03
78	0.02	0.06	0.00	0.04	0.03	0.02	0.02	0.04
80	0.02	0.07	− 0.01	0.04	0.03	0.02	0.02	0.04
82	0.01	0.09	− 0.02	0.05	0.03	0.02	0.02	0.03
84	0.01	0.12	− 0.03	0.05	0.02	0.03	0.01	0.03
86	0.00	0.13	− 0.05	0.04	0.02	0.02	0.01	0.02
88	0.00	0.08	− 0.03	0.03	0.00	0.03	− 0.01	0.01
90	− 0.04	0.16	− 0.09	0.02	− 0.02	0.05	− 0.04	0.00
92	− 0.06	0.17	− 0.11	0.00	− 0.05	0.06	− 0.07	− 0.03
94	− 0.08	0.16	− 0.13	− 0.02	− 0.07	0.06	− 0.09	− 0.05
96	− 0.09	0.13	− 0.13	− 0.04	− 0.08	0.06	− 0.10	− 0.06
98	− 0.09	0.12	− 0.13	− 0.05	− 0.09	0.07	− 0.11	− 0.06
100	− 0.09	0.19	− 0.15	− 0.02	− 0.07	0.07	− 0.10	− 0.05
Hip joint moment in sagittal plane								
2	− 0.04	0.16	− 0.09	0.02	− 0.02	0.10	− 0.06	0.01
4	− 0.11	0.20	− 0.18	− 0.04	− 0.09	0.18	− 0.15	− 0.03
6	− 0.20	0.24	− 0.28	− 0.12	− 0.16	0.25	− 0.24	− 0.07
8	− 0.26	0.29	− 0.36	− 0.17	− 0.20	0.33	− 0.32	− 0.09
10	− 0.31	0.35	− 0.42	− 0.19	− 0.22	0.42	− 0.37	− 0.08
12	− 0.34	0.38	− 0.47	− 0.21	− 0.23	0.49	− 0.40	− 0.06
14	− 0.35	0.40	− 0.49	− 0.21	− 0.23	0.54	− 0.41	− 0.04
16	− 0.34	0.42	− 0.48	− 0.19	− 0.19	0.54	− 0.38	0.00
18	− 0.30	0.42	− 0.44	− 0.16	− 0.14	0.52	− 0.32	0.04
20	− 0.24	0.41	− 0.38	− 0.10	− 0.10	0.49	− 0.27	0.07
22	− 0.16	0.40	− 0.30	− 0.03	− 0.04	0.46	− 0.20	0.12
24	− 0.09	0.40	− 0.22	0.05	0.02	0.44	− 0.13	0.17
26	− 0.02	0.40	− 0.16	0.11	0.08	0.41	− 0.06	0.22
28	0.04	0.41	− 0.10	0.18	0.14	0.40	0.00	0.28
30	0.09	0.43	− 0.06	0.23	0.20	0.41	0.06	0.34
32	0.14	0.45	− 0.01	0.29	0.27	0.40	0.13	0.41
34	0.18	0.47	0.02	0.33	0.32	0.42	0.18	0.47
36	0.22	0.48	0.06	0.38	0.36	0.42	0.21	0.50
38	0.26	0.49	0.09	0.42	0.40	0.43	0.25	0.55
40	0.30	0.51	0.13	0.47	0.40	0.45	0.25	0.56
42	0.36	0.54	0.18	0.54	0.41	0.47	0.25	0.58
44	0.42	0.58	0.23	0.62	0.41	0.49	0.24	0.58
46	0.49	0.61	0.28	0.70	0.42	0.51	0.24	0.59
48	0.56	0.64	0.34	0.78	0.43	0.53	0.24	0.61
50	0.62	0.66	0.40	0.84	0.45	0.57	0.25	0.65
52	0.68	0.70	0.44	0.91	0.51	0.63	0.29	0.72
54	0.73	0.82	0.45	1.00	0.56	0.72	0.31	0.81
56	0.76	0.95	0.44	1.08	0.60	0.83	0.32	0.89
58	0.75	1.00	0.41	1.08	0.66	0.93	0.33	0.98
60	0.67	0.99	0.34	1.01	0.68	0.93	0.36	1.00
62	0.56	0.94	0.25	0.88	0.63	0.88	0.32	0.93
64	0.44	0.84	0.16	0.72	0.53	0.76	0.27	0.80
66	0.31	0.72	0.06	0.55	0.41	0.63	0.19	0.62
68	0.16	0.60	− 0.04	0.36	0.27	0.55	0.08	0.46
70	0.05	0.55	− 0.14	0.24	0.15	0.41	0.01	0.29
72	− 0.02	0.54	− 0.20	0.16	0.07	0.29	− 0.03	0.16
74	− 0.04	0.49	− 0.20	0.13	0.03	0.22	− 0.05	0.11
76	− 0.03	0.28	− 0.12	0.06	− 0.01	0.11	− 0.05	0.03
78	0.00	0.13	− 0.05	0.04	− 0.02	0.06	− 0.04	0.01
80	0.02	0.10	− 0.01	0.05	0.00	0.06	− 0.02	0.02
82	0.04	0.12	0.00	0.08	0.01	0.06	− 0.01	0.03
84	0.06	0.17	0.00	0.11	0.03	0.07	0.01	0.06
86	0.07	0.21	0.00	0.14	0.04	0.09	0.01	0.07
88	0.04	0.25	− 0.05	0.12	0.06	0.12	0.02	0.11
90	0.11	0.23	0.04	0.19	0.10	0.14	0.05	0.15
92	0.14	0.20	0.07	0.20	0.13	0.16	0.08	0.19
94	0.14	0.15	0.09	0.19	0.16	0.18	0.10	0.22
96	0.12	0.16	0.07	0.18	0.17	0.18	0.11	0.23
98	0.13	0.14	0.08	0.18	0.16	0.17	0.10	0.22
100	0.10	0.16	0.04	0.15	0.13	0.16	0.08	0.19

The ankle joint moment curves for both groups showed a similar pattern, starting with a mild dorsiflexion and progressing to plantar flexion during almost all of the stance phase. The PN group had a higher magnitude of their peak M_A1_ compared to the PFA group. For the knee joint moment curves, both groups showed a similar pattern with an extension second peak of knee moment M_K2_. The PN group reached their second peak M_K2_ earlier compared to the PFA group, with a higher magnitude of M_K2_ compared to the PFA group. The hip joint moment curves for both groups showed a similar pattern with a flexion third peak of hip moment M_H3_. The PFA had a lower magnitude of M_H1_ compared to the PN group. The PN group had a higher magnitude of M_H2_ compared to the PFA group (Table 3, Fig. 5).

## Discussion

This study is part of a comprehensive gait analysis in patients with PFA secondary to PN, compared to PN patients alone. The focus is on the changes in the sagittal plane kinematics and moments of lower limb joints that could cause the compensatory mechanisms in spatiotemporal characteristics, kinetics, and loading pattern that were revealed in our previous work [21, 22]. The study findings revealed that, compared to published normative data, both PFA and PN patients walk at a slow speed with alterations of sagittal plane kinematics and moments mainly during late stance [28–32]. In terminal stance both groups displayed a higher ankle dorsiflexion, an extension lag of the knee joint, and a reversed second peak of the knee moment. In preswing both groups showed a reduced plantar flexion up to loss in the PFA group and a reduced and delayed peak of the ankle plantar moment.

During terminal stance, the advancement of the tibia is primarily controlled by the calf muscles, which virtually lock the ankle joint, allowing both the tibia and foot to roll forward on the forefoot rocker [33, 34]. However, the study results suggests that the ankle joint, rather than the forefoot rocker, served as a pivot, and the tibia was accelerated forward over the ankle as the COM progressed due to the momentum of the swinging contralateral limb. It’s possible to hypothesize that the observed increase in dorsiflexion beyond the normal range is indicative of less eccentrically loaded calf muscles, which was supported by the observed reduction and delay in the plantar flexion peak of the ankle moment. Consequently, the tibia wasn’t adequately controlled over the foot, resulting in the recorded higher dorsiflexion ROM and prolonged terminal stance in both groups. As a result, the femur was forced to roll over the unstable tibia in order to maintain the knee extension and decelerate the drop of the center of gravity and the vertical collapse of the body by the generation of a knee extension moment during terminal stance. These alterations were identified by a reversed second peak of the knee moment, which was a knee extension moment instead of the supposed knee flexion moment during late stance, and a delayed third peak of the knee ROM.

During preswing, the plantar flexion ROM of the ankle joint arises primarily from the elastic recoil of the highly eccentrically loaded calf muscles during terminal stance [35, 36]. Therefore, the recorded reduction in the plantar flexion during the preswing for both groups could be the logical continuity of their altered kinematics and moment during the terminal stance subphase. In the PFA group, the reduction was significantly greater, limiting plantar flexion as the ankle remained in a few degrees of dorsiflexion. However, further research with a comprehensive assessment of muscle control, strength, and flexibility is needed to establish such a possible causative factor, as the current study did not assess the flexibility and strength of the calf muscles.

Previous studies have reported that normal ankle kinematics and moment includes approximately 10° of dorsiflexion during terminal stance occurring at 50% of the gait cycle, approximately 15° of plantar flexion during preswing, and a maximum plantar flexion moment of approximately 1.4 (N.m/kg) occurring during terminal stance at 47% of the gait cycle [28–32]. Our findings are consistent with previous studies that showed an increase in ankle dorsiflexion during terminal stance for both the PFA and PN groups [37–40]. Similarly, the observed delay of the dorsiflexion peak for PFA and PN groups is consistent with previous studies [38–40]. However, there was no significant difference between both groups regarding the dorsiflexion peak. Additionally, our findings are consistent with previous studies that showed reduction in the plantar flexion ROM during preswing for the PFA group [38, 41], and for the PN group [40]. However, unlike previous studies, we observed a complete loss of plantar flexion in the PFA group. The observed reduction in the peak of the plantar flexion moment for the PFA group is consistent with previous studies [20, 38, 39, 41, 42], and the results for the PN group were consistent with a previous study [40].

Normally, the second peak of the knee moment is reported to be a flexor moment during late stance [28–32]. The observed reversing in the second peak of the knee moment in both groups was consistent with a previous study [41]. The recorded extension moment during terminal stance identifies an increased demand on the quadriceps, which delays the third peak of the knee ROM. Normally, the third peak previously was reported to be reached at 70% of the GC [28–30]. This delay is consistent with a previous study [38].

With regard to the hip joint, normally, the hip extension during terminal stance and hip flexion during late swing are reported to be 10° and 25° respectively [28–30], Both groups showed increased hip extension during terminal stance and decreased hip flexion during late swing, which could identify the suggested compensatory mechanisms taken by the ankle and knee joints in both groups, but there was no significant difference between both groups. The reduced hip flexion during late swing could be the logical consequence of the increased hip extension and the prolonged terminal stance subphase, which is consistent with previous studies [38, 41]. Interestingly, hip extension was reported to be reduced in the study by Mueller et al. [41], which contradicts our findings of increased hip extension.

The contradiction with the study by Mueller et al. [41], regarding hip extension and loss of plantar flexion, and with the studies by Dillon [38], regarding loss of plantar flexion in the PFA group, could be due to the differences in the test procedure, as our participants walked barefoot and didn’t use orthotics or prosthetics while theirs did, assuming that these gait compensations could be an involuntary attempt to protect their forefoot.

Walking speed is the result of both gait kinematics and kinetics. Normally, the average value of the self-preferred speed was reported to be 1.334 (m/s) [43, 44]. Both PN and PFA walked slowly with no significant difference between the two groups. The results were consistent with those of the previous study that compared similar groups [45]. The observed reduction in walking speed for the PN group was consistent with previous studies [40, 46–48]. The results from the PFA group were consistent with previous studies [19, 39, 49–52].

### Limitations

Besides the difficulties with recruiting an adequate number of patients with PFAs that were previously detailed in the previous work [21], not including joint moment in the MANOVA analysis was another limitation of the current study. The reason for the non-inclusion was the large variability in the shape of the curves among patients. The large variability could be attributed to walking at a slow speed [53].

## Conclusion

Individuals of both groups with PFA due to PN or PN alone showed similar alterations in the sagittal plane kinematics and moments of the lower limb joints, they tend to walk cautiously with excessive dorsiflexion throughout the stance phase. The late stance phase was the most affected by higher ankle dorsiflexion in terminal stance, reduced plantar flexion up to the loss in the PFA group during preswing, and reduced and delayed peak of the ankle plantar moment. We suggest that they compensated the reduction in the ankle plantar moment with shifting the knee moment into extension moment. Our results also suggest that PN, not PFA, may be the primary cause of the gait alterations, and the PFA surgery only worsens the compensatory mechanisms.

Our findings emphasize the need for early intervention and a multidisciplinary approach to manage the systemic disease and prevent the need for amputation. Further research of longitudinal design, which allows for causal inference, would be recommended to confirm our findings and develop more effective interventions for improving gait function and quality of life for individuals with PN and PFA.

## Data Availability

The corresponding author will provide the datasets used and/or analyzed for the current work upon reasonable request.

## Abbreviations

PFA: Partial foot amputation
PN: Peripheral neuropathy
ROM: Range of motion
GRF: Ground reaction force
MTP: Metatarso phalangeal joint
3DMA: Three dimensional motion analysis
M: Moment
COP: Center of pressure
COM: Center of mass
